# Fluorescent single-stranded DNA-binding protein from *Plasmodium falciparum* as a biosensor for single-stranded DNA

**DOI:** 10.1371/journal.pone.0193272

**Published:** 2018-02-21

**Authors:** Liisa T. Chisty, Daniela Quaglia, Martin R. Webb

**Affiliations:** The Francis Crick Institute, London, United Kingdom; University of Iowa, UNITED STATES

## Abstract

Single-stranded DNA (ssDNA) is a product of many cellular processes that involve double-stranded DNA, for example during DNA replication and repair, and is formed transiently in many others. Measurement of ssDNA formation is fundamental for understanding many such processes. The availability of a fluorescent biosensor for the determination of single-stranded DNA provides an important route to achieve this. Single-stranded DNA binding proteins (SSBs) protect ssDNA from degradation, but can be displaced to allow processing of the ssDNA. Their tight binding of ssDNA means that they are very good candidates for the development of a biosensor. Previously, the single stranded DNA binding protein from *Escherichia coli*, labeled with a fluorophore, (DCC-EcSSB) was developed and used for this purpose. However, the multiple binding modes of this protein meant that interpretation of DCC-EcSSB fluorescence was potentially complex in terms of determining the amount of ssDNA. Here, we present an improved biosensor, developed using the tetrameric SSB from *Plasmodium falciparum* as a new scaffold for fluorophore attachment. Each subunit of this tetrameric SSB was labeled with a diethylaminocoumarin fluorophore at a single site on its surface, such that there is a very large, 20-fold, fluorescence increase when it binds to ssDNA. This adduct can be used as a biosensor to report ssDNA formation. Because SSB from this organism has a single mode of binding ssDNA, namely 65–70 bases per tetramer, over a wide range of conditions, the fluorescent SSB allows simple quantitation of ssDNA. The binding is fast, possibly diffusion controlled, and tight (dissociation constant for DCC-PfSSB <5 pM). Its suitability for real-time assays of ssDNA formation was demonstrated by measurement of AddAB helicase activity, unwinding double-stranded DNA.

## Introduction

Many cellular processes involving DNA, such as replication, transcription and repair rely on separating double-stranded DNA (dsDNA) to form single-stranded DNA (ssDNA). Measurement of ssDNA formation is fundamental for understanding many such processes, in particular the activity of the large number of helicases that catalyze the strand separation using the energy of ATP hydrolysis. DNA helicase activity can be assayed directly by measuring either the formation ssDNA or loss of dsDNA. Several assays have been developed, mostly based on fluorescence of intercalating dyes, whereby the dye binds to dsDNA preferentially over ssDNA [1, 2] and bound dye is depleted as ssDNA forms. However, such depletion assays modify the substrate, here dsDNA, and so may alter the activity being measured. It is often preferred to measure the product ssDNA, as the measurement is less likely to interfere with the activity.

One approach, particularly useful for product measurement, is to use a fluorescent, reagentless biosensor. Such probes have a recognition element, such as a protein, that binds specifically the analyte of interest, in this case ssDNA. There is also a fluorophore in the biosensor that responds to this binding to give the signal. This approach can produce real-time, sensitive, rapid measurements *in situ* [3]. Single-stranded DNA-binding protein (SSB) has lent itself to such measurements, as it binds tightly and quantitatively to ssDNA, completely wrapping and sequestering long stretches of ssDNA. Indeed, it has a major function in the cell of protecting ssDNA from degradation or modification. Previously, a fluorophore-SSB adduct was developed as a biosensor to assay ssDNA, using SSB from *Escherichia coli* (EcSSB) and diethylaminocoumarin as the fluorophore [4]. This adduct, DCC-EcSSB, gave ~6-fold increase in fluorescence, when fully bound by ssDNA and has been used to assay ssDNA formation in real time as helicases unwind dsDNA [5, 6]. More recently, *Bacillus subtilis* SSB, attached to an Alexa-dye, has also been used [7].

In bacteria, SSBs generally have similar structures and are typically obligate dimers or tetramers. At most 65-70-base length of ssDNA wraps around tetrameric SSB in a “baseball seam” manner. Although binding is very rapid, perhaps diffusion controlled, and tight with a nanomolar dissociation constant [8], application of DCC-EcSSB to ssDNA assays has been complicated by the existence of multiple binding modes of ssDNA to this *E*. *coli* protein [9, 10]. The length of ssDNA binding to each SSB tetramer is dependent on experimental conditions, particularly the ratio of SSB to DNA, their concentrations and the ionic strength or salt conditions [8]. Under low salt conditions, the preferred mode has only ~35 bases of DNA binding per SSB tetramer [8, 10].

With DCC-EcSSB this change in binding mode produces a change in fluorescence enhancement [4]. The preferred, thermodynamically most stable binding mode may change during the course of a real-time assay as relative concentrations change or within a set of related assays as total concentrations differ among the set. Examples of how this is exhibited can be garnered from previous work measuring dT_70_ binding to DCC-EcSSB. At high salt, a titration of dT_70_ into DCC-EcSSB produced a linear, monotonic increase in fluorescence until the ratio of dT_70_:DCC-EcSSB-tetramer was one, suggesting each DNA molecule binds similarly to the protein until the latter is saturated (Fig 3 in reference [4]). In contrast at low salt, the titration response was not monotonic, as the binding changed with the ratio of DNA to protein. At low ratio, 35-base binding dominates with a smaller fluorescence enhancement. The binding time course also can show the change in binding mode (Fig 2 in reference [8]). After an initial increase in fluorescence as dT_70_ bound to excess DCC-EcSSB, there was a slower second phase decrease as the binding mode changed. The amplitude of this rearrangement increased as the concentrations increased, favouring 35-base mode binding. This change in binding mode complicates calibration of the fluorescence signal and limits the types of assays that are possible.

To overcome these complicating factors, a similar SSB from a different organism (*Plasmodium falciparum*) has been investigated that leads to simpler assays for quantification of the formation of ssDNA, due to simpler binding modes. This SSB has been characterized previously in terms of both its structure and its DNA binding properties [11–13]. The crystal structure of PfSSB (24.5 kDa subunit) DNA-binding domain showed that it is highly similar to bacterial SSBs and specifically with EcSSB, although DNA binds with opposite polarity [13]. Major differences occur in the intrinsically disordered region at the C-terminus, which is 65 amino acids long in EcSSB and >80 amino acids long in PfSSB [11, 12, 14, 15]. This region is not visible in the crystal structures of either species’ SSB but includes the domain responsible for the cooperative binding of SSB to ssDNA and for the interaction with other proteins involved in the DNA replication [12, 16, 17]. In *E*. *coli*, this region is formed of a 56 amino acid intrinsically disordered (ID) linker and a 9 amino-acid acidic tip and it appears that the cooperative binding is mainly due to 35-base binding mode [10, 18]. Interestingly for EcSSB, replacing the linker region between the acid tip and the DNA-binding domain with that from PfSSB eliminates the 35-base binding mode and, hence, cooperativity. In PfSSB, the acid tip is similarly nine amino acids. However, importantly for potential use as a biosensor, ssDNA binding to PfSSB is simpler than with EcSSB. It has been shown that the 65-70-base binding mode predominates over the 35-base binding mode under a wide range of conditions [11].

Here we report a new probe for ssDNA, developed by forming an adduct of PfSSB with diethylaminocoumarin fluorophore. Our strategy was to survey several SSB-fluorophore adducts, prepared through labeling single cysteines at various locations on the surface of each subunit, close to, but not within, the binding channel. The cysteine was then uniquely labeled with a thiol-reactive dye. The resulting adducts give a fluorescence signal of various sizes on ssDNA binding.

The best combination of fluorophore, labeling position and protein construct was chosen, based on the magnitude of the fluorescence change on binding ssDNA and on the ability of the adduct to bind ssDNA in a single mode over a wide range of solution conditions. The best combination was a diethylaminocoumarin with which the protein was labelled in position C93. This adduct shows tight and rapid binding of ssDNA with a maximum fluorescence change of 20-fold. Regardless of the salt concentration in the buffer and the ratio of SSB to ssDNA, only one primary binding mode to ssDNA was detected. The ability of this adduct to monitor ssDNA formation in a real-time assay is also confirmed by a helicase activity assay.

## Materials and methods

### Materials

IDCC (N-[2-(iodoacetamido)ethyl]-7-diethylaminocoumarin-3-carboxamide), 6-IATR (6-iodoacetamidotetramethylrhodamine) and 5-IATR (5-iodoacetamidotetramethylrhodamine) were gifts from J. Corrie (NIMR, London, U.K.) [19–21]. dT_35_, dT_70,_ poly(dT) and other chemicals were from Sigma-Aldrich unless otherwise stated. Poly(dT) has an average length ~500 bases and in all measurements, its concentration was calculated in terms of 70-base units, approximating to the SSB binding sites. AddAB helicases (wild type from *Bacillus subtilis* and helicase-inactive mutant, AddA^*H*^B(A-K36A)) were gifts from Mark Dillingham (University of Bristol, U.K.) [22].

Biotin-TEG-DNA, linear dsDNA of different lengths but without Chi-sequences, for the AddAB assay, was prepared by PCR from the pCERoriD 4907 bp plasmid [6] and amplifying the sequences using 5’-biotinylated forward primer (5’bio-TEG-GGG TAT TGT TTG TTC CCT GAG CGC G 3’) and various reverse primers (585 bp: 5’-GTC TGC CTC CAC CGC GGC CAC G 3’, 985 bp: 5’- CTG TGC CAT GAC GGA GGA TGA TG 3’, 1216 bp: 5’-AGG AAA ACC GGT ACA GAA CTG CA 3’, and 1458 bp: 5’-CGG CAT ACC TCA GTG GCG TGG AG 3’). Bio-DNAs were gel purified using Qiagen Gel Extraction kit.

### *P*. *falciparum* SSB and choice of labeling position

Native *P*. *falciparum* SSB is transcribed and translated from nuclear DNA with a 76-amino-acid, apicoplast-localization sequence (ALS) that is cleaved off once delivered to the apicoplast [23]. PfSSB was expressed without the N-terminal ALS (76 amino acids) but amino acids are numbered for the full-length protein, that is the first amino acid in the expressed protein is M77. This truncated wild-type gene expressing the 208 amino acid PfSSB but with the G103C mutation already present (MNEKSLNKIM LIGRVGCEPD IKILNGCDKV ATFSLATNEF WRDRNTNELK SKTDWHRIVV YDQNIVDLID KYLRKGRRVY VQGSLHTRKW HTNDMNSQPK QITEIILSYN KGDLIFLDDK RNFNQRNNSN NINSENQQHI NNEHINNNNI NNGNDFMPLN SNDKIIEDKE FTDRLDDNNE ENNFQSNSET FDKQEGIYDK MNVQEFEE) was from GenScript (The Biology CRO, U.S.A.). Other variants were mutated from this gene.

Wild-type PfSSB has a single cysteine C93 that is located on the surface of PfSSB and can be expected to be accessible to labeling using iodoacetamide or maleimide chemistry. Other sites were also tested, by mutation of the wild-type cysteine to alanine and then introducing new surface cysteines. The positions were selected, based on the crystal structures (PDB ID: 3ULP), such that the changes were not in the ssDNA binding groove, but also by comparison with the similar structure of EcSSB (PDB ID: 1EYG, 1QVC,1SRU, 4MZ9) and its successful labeling locations [4, 11, 14, 24]. Constructs were prepared using a QuikChange mutagenesis kit and protocol (Stratagene) and primers as in S1 Table and are summarized in Table 1.

**Table 1:** Signal changes of adducts of PfSSB variants and fluorophore dyes.

PfSSB variant	Labeling reagent	Fluorescence ratio ± dT_70_
Wild type	IDCC	20.1 ±0.1
Wild type	5-IATR	9.1 ±0.2
Y156R	IDCC	12.3 ±0.07
C93A G103C	IDCC	3.5 ±0.08
C93A G102C	IDCC	1.8 ±0.09
C93A W166C	5-IATR	12.8 ±0.5
C93A G103C	6-IATR	3.5 ±0.4

Excitation and emission spectra were measure with 250 nM PfSSB adduct in the presence and absence of 595 nM dT_70_. The signal change (+/- standard error from 3 replicates) is given as a ratio of fluorescence with ssDNA divided by fluorescence without ssDNA at the wavelengths of maximum excitation and emission: 432 nm and 475 nm for diethylaminocoumarin and 556 nm and 577 nm for tetramethylrhodamine.

Wild-type SSB and cysteine mutants were expressed from pET22b in C41(DE3) *E*. *coli* cells using the T7 promoter system (Lucigen, WI, U.S.A.). The PfSSB was prepared using the protocol for EcSSB [4] but omitting the polymin P precipitation. Its concentration was calculated from the absorbance at 280 nm, using an extinction coefficient of 95800 M^-1^ cm^-1^ for the tetramer [13].

### Fluorophore labeling of *Pf*SSB

Typically, 10 mg of each PfSSB variant was labeled using iodoacetamide-thiol chemistry and all labeling steps were at room temperature. First, PfSSB was incubated with 10-fold excess dithiothreitol (DTT) over PfSSB subunits for 20 min. DTT was removed by elution through a PD-10 column (GE Healthcare) with the labeling buffer (20 mM Tris·HCl pH 7.5, 1.0 mM EDTA, 500 mM NaCl, 20% (v/v) glycerol). IDCC was added in 2.5-fold excess over ~100 μM PfSSB subunits and incubated 2 h with end-to-end stirring. Excess IDCC was removed by incubating with sodium 2-sulfanylethanesulfonate (MESNA) in 10-fold excess for 20 min. The mixture was passed through a 0.2 μm syringe filter HT Tuffryn membrane (Sigma-Aldrich) before passing through a 10 ml P4 gel-filtration column (Biorad), pre-equilibrated with the labeling buffer. Labeled PfSSB was concentrated using an Amicon Ultracentrifuge filter unit with a molecular weight cut-off of 10 kDa. The concentration of IDCC-labeled PfSSB and the ratio of the coumarin to protein was determined from the absorbance spectrum: at 280 nm both fluorophore and protein contribute, at 430 nm only the fluorophore., The extinction coefficient for IDCC is 7470 M^-1^ cm^-1^ at 280 nm 44800 M^-1^ cm^-1^ at 430 nm and assumed to be the same on attachment to the protein.[19]. Typically, the ratio of label to protein was 1 +/- 10%. Mass spectrometry of DCC-PfSSB gave a mass of 24,917±5 Da, agreeing with the theoretical mass (24,914 Da), assuming the N-terminal methionine had been cleaved. The labeled PfSSB was stored at -80°C.

### Absorbance and Fluorescence measurements

Absorbance was measured on a Jasco spectrophotometer at 20°C. Fluorescence was measured at room temperature on a Cary Eclipse spectrofluorimeter (Varian Inc.) with a xenon lamp using 3-mm pathlength quartz cells.

Titrations with diethylaminocoumarin-labeled SSBs were done by excitation at 433 nm and emission at 473 nm (both slits at 2.5 nm). For titrations to determine the relative affinities of labeled and unlabeled SSB, dT_70_ was titrated into a solution containing equimolar labeled and unlabeled PfSSB and the data was fitted between the origin and saturation of both proteins at the break point using the equation below:

$$\Delta Fluorescence=\left(F_{max}–F_{min}\right)\left(-b+sqrt\left(b^{2}+4\left(K-1\right)PL\right)\right)/\left(2L\left(K-1\right)\right)$$ Eq 1

Where *F*_min_ = fluorescence in the absence of DNA, *F*_max_ = fluorescence of DCC-PfSSB·ssDNA, *b = KX + P + L–KP*, *X* = [unlabeled PfSSB], *L* = [DCC-PfSSB], *P* = [total ssDNA], *K* = ratio of *K*_d_ values for the two SSB species (labeled/unlabeled).

Titrations at very low [DCC-PfSSB] were fitted to a quadratic binding equation:

$$\Delta Fluorescence=\left(F_{max}–F_{min}\right)\left(K_{d}+P+L-sqrt\left({\left(K_{d}+P+L\right)}^{2}-4LP\right)\right)/\left(2L\right)$$ Eq 2

Where *F*_min_ = fluorescence in the absence of DNA, *F*_max_ = fluorescence of DCC-PfSSB·ssDNA, *L* = [DCC-PfSSB], *P* = [total ssDNA] and *K*_d_ is the dissociation constant.

The fluorescence quantum yields were measured as previously described [25] using Coumarin 343 as a standard with a value of 0.63 [26].

The stopped-flow apparatus was a HiTech scientific SF61MX stopped-flow (TgK Scientific Ltd., U.K.) with a mercury-xenon lamp. All measurements with DCC-PfSSB and DCC-EcSSB had the excitation at 436 nm and fluorescence emission was measured with a 455 nm cut-off filter (Schott glass). The concentrations given are final concentrations for the components in the mixing chamber. Measurements were repeated as separate experiments three times unless otherwise stated. Kinetic Studio software (TgK Scientific Ltd., U.K.) was used to record and fit the data. Time courses shown in figures are of representative single measurements. Further data fitting was done using Grafit 7 (Erithacus Software Ltd., U.K.) [27]. Plots of rate constants show averages with standard errors from da minimum of three acquisitions. Values of parameters derived from such plots are given with (+/-) standard errors

All fluorescence and kinetic measurements (without helicase) were at 20°C in high salt buffer (25 mM Tris·HCl pH 7.5, 200 mM NaCl and 10 μM BSA) except when stated, in which case the low salt buffer had 20 mM NaCl instead of 200 mM.

### Helicase assays

The AddAB helicase assay for unwinding linear dsDNA was performed on the stopped-flow apparatus at 37°C in buffer containing 25 mM Tris·acetate (pH 7.5), 2 mM magnesium acetate, 1 mM DTT and 10 μM BSA. 1 nM of bio-DNA was incubated with 10 nM streptavidin for 15 min at room temperature. To this 8 nM of AddAB wild type helicase was added and incubated 1 min at room temperature. The mixture was moved to a stopped-flow syringe and incubated for further 1 min at 37°C before rapidly mixing with 200 nM DCC-PfSSB, 100 nM AddA^*H*^B (trap) and 2 mM ATP.

## Results

### Survey of different fluorophore-PfSSB adducts

Several SSB-fluorophore adducts were prepared, as described above, to determine a suitable combination of cysteine position on the protein surface (and hence fluorophore location) to give a suitable signal on ssDNA binding. Proteins were labeled mainly with diethylaminocoumarin through iodoacetamide (IDCC) or with tetramethylrhodamine iodoacetamide, coupling with the cysteine thiol (Table 1). Protein-fluorophore adducts were surveyed initially under one set of high salt conditions with dT_70,_ a simple model for complete ssDNA binding. As the binding site of PfSSB has been measured as ~65–70 bases [11, 13], it was expected that the binding stoichiometry would be one dT_70_ per PfSSB tetramer. The highest fluorescence change was observed with PfSSB, in which C93 (the wild-type cysteine) was labeled with IDCC (Table 1). In contrast, labeling on the loop that was used with EcSSB (G26C in EcSSB aligns with G102C in PfSSB) gave relatively low responses. Hence, the wild-type protein, labeled with IDCC and hereafter termed DCC-PfSSB, was chosen for further study.

### Fluorescence properties of DCC-PfSSB

**Fig 1:**
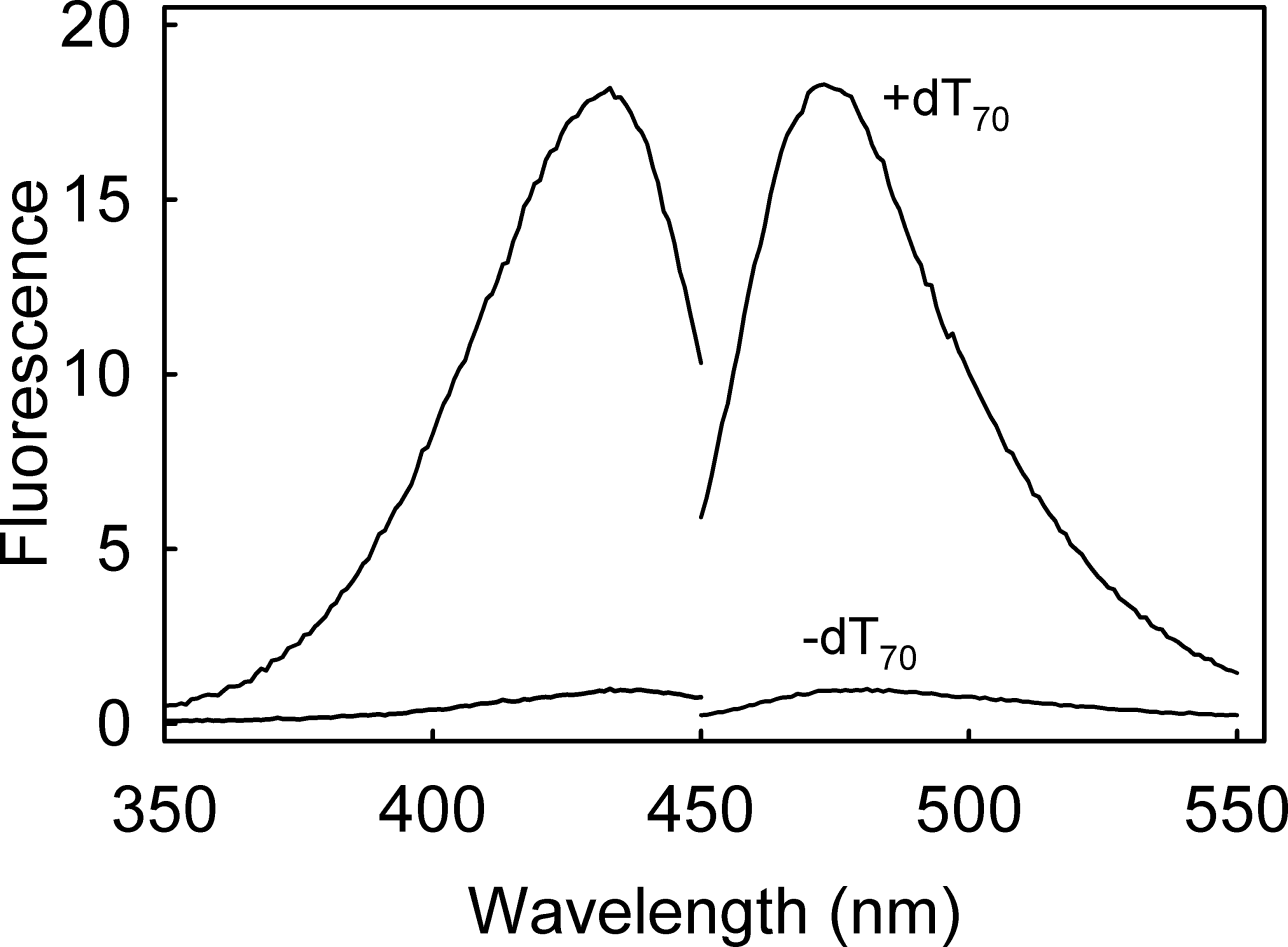
Fluorescent spectra of DCC-PfSSB. Excitation and emission spectra of 250 nM DCC-PfSSB with and without 595 nM dT_70_ in high salt buffer. The excitation spectra had emission at 475 nm; emission spectra had excitation at 432 nm. The spectra were normalized to one at the emission maximum in the absence of DNA.

The change in fluorescence spectra of DCC-PfSSB on binding dT_70_ is shown in Fig 1, giving a 20-fold increase in intensity in the high salt buffer. The fluorescence quantum yield for DCC-PfSSB was determined under these conditions as 0.01 without dT_70_, and 0.11 when saturated with dT_70_. In comparison, the same fluorophore attached to a small molecule, sodium 2-sulfanylethanesulfonate (MESNA) had a quantum yield of 0.02 in the same conditions, suggesting that fluorescence is quenched in the apoprotein relative to free fluorophore, in contrast to the enhancement in the SSB.ssDNA complex.

### DNA binding properties of DCC-PfSSB

**Fig 2:**
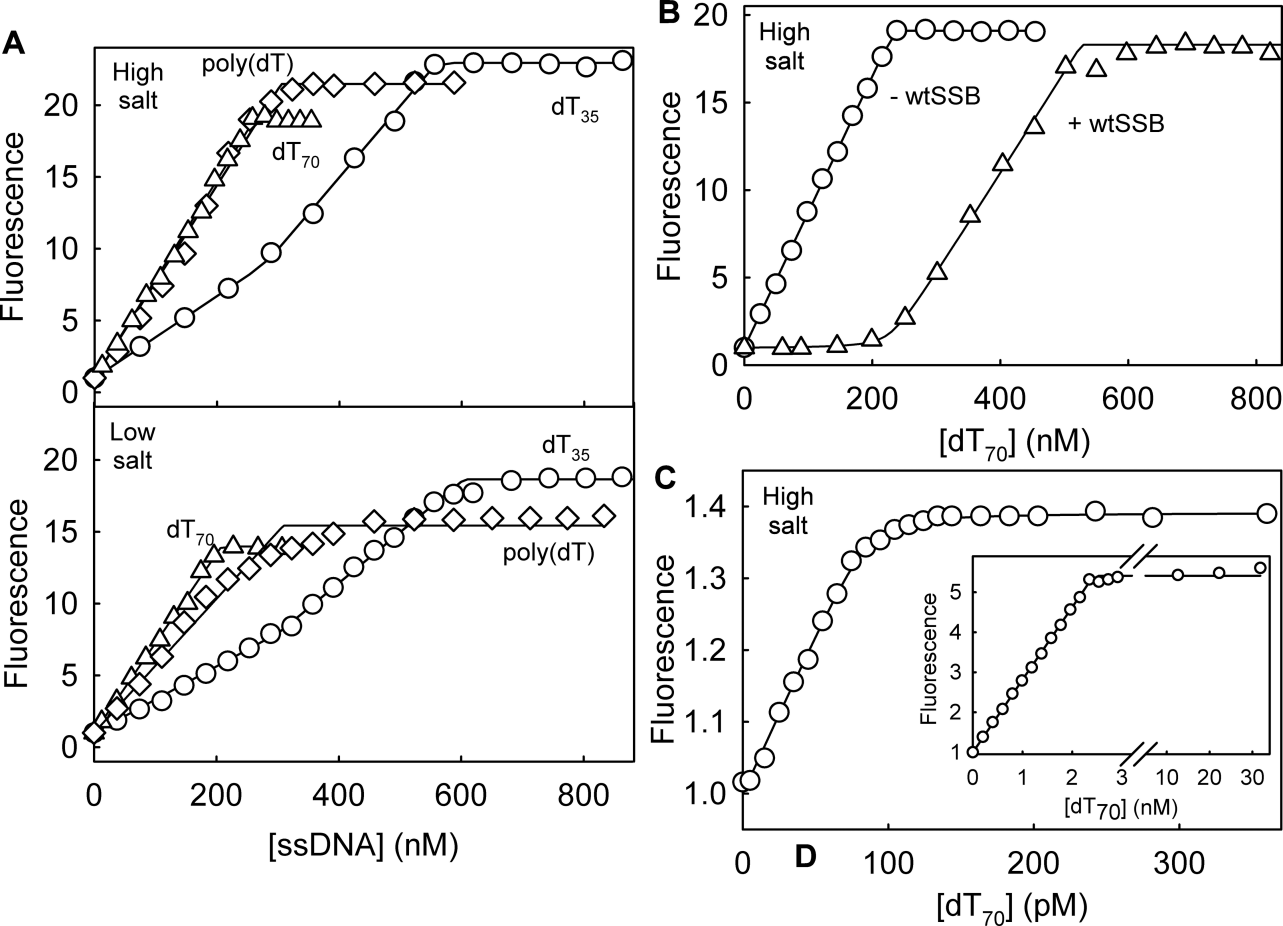
Titrations of different ssDNA lengths with DCC-PfSSB. (A) Titrations of dT_70_ (triangles), dT_35_ (circles) and poly(dT) (diamonds) into 250 nM DCC-PfSSB. Upper panel were in high salt, lower panel in low salt buffer. Data were fit for tight binding, a linear increase followed by a constant fluorescence with floating breakpoint, or in the case of dT_35_, two linear increases. (B) dT_70_ titrations into 250 nM DCC-PfSSB in high salt buffer with and without equimolar unlabeled PfSSB. Data were fit to Eq 1, as described in the Materials and Methods. (C) Titrations with dT_70_ at low DCC-PfSSB in high salt buffer. At 2.5 nM (main panel) the data were fit to a linear increase followed by a constant fluorescence. At 125 pM (inset), the data from a single titration were fit to a quadratic binding equation (Eq 2), giving a *K*_d_ of 1.2 ± 0.5 pM. No correction to amplitudes was made for background scatter, which is the likely cause of the apparently lower fluorescence change.

A titration of DCC-PfSSB at high salt showed a linear increase in fluorescence as dT_70_ was added, followed by a sharp break to a constant fluorescence when dT_70_:DCC-PfSSB (as tetramer) was 1:1 (Fig 2A). This indicated that the binding of DCC-PfSSB was stoichiometric under these conditions: measurements are described below to attempt to measure the dissociation constant

When a similar titration was performed with dT_35_ (Fig 2A), the plot had two linear phases of fluorescence increase, followed by a sharp break to constant fluorescence. As the break points represent approximately 1:1 and 2:1 dT_35_: tetramer, his presumably corresponds to sequential binding of two dT_35_ molecules to each tetramer, so that the protein is saturated with two dT_35_ molecules per tetramer. The fluorescence change on binding the first dT_35_ was slightly lower than with the second. A titration with poly(dT) (Fig 2A) showed a linear fluorescence increase similar to dT_70_, suggesting that when multiple SSBs are bound along the DNA, then the fluorescence response is similar to having a single DCC-PfSSB.dT_70_ complex. In both cases (dT_35_ and poly(dT)), the overall fluorescence change was similar to that with dT_70_.

The effect of IDCC labeling on the PfSSB affinity to ssDNA was tested by performing competition titrations of dT_70_ with DCC-PfSSB in the presence of unlabeled PfSSB (Fig 2B). There was a low increase in fluorescence at the beginning of titration where the amount of available ssDNA was low compared to the end of titration (Fig 2B). This indicated that at low DNA concentrations, the limiting ssDNA bound tighter, and so preferentially, to the unlabeled PfSSB. As more DNA was added and unlabeled SSB became saturated, the DNA bound to the DCC-PfSSB and the fluorescence increased. The final breakpoint was approximately at the sum of the concentrations of the two SSBs. Fitting the dT_70_ titration curve with unlabeled and labeled PfSSB using Eq 1 gave a limit on ratio of the dissociation constants with the unlabeled protein binding >25-fold tighter.

Titrations were also performed with much lower DCC-PfSSB concentration (125 pM and 2.5 nM) with the aim of getting a dissociation constant directly (Fig 2C). At 2.5 nM DCC-PfSSB the binding of DCC-PfSSB to dT_70_ remained stoichiometric: the response was linear up to a sharp break point at a DCC-PfSSB:dT_70_ of ~1. At 125 pM DCC-PfSSB, the titration was still linear, but deviated from this at close to saturation. The data were fitted as described in Fig 2C, suggesting a *K*_d_ in the low picomolar range.

### Effect of changing the salt concentration on SSB Binding to ssDNA

Titrations were also done with different lengths of oligonucleotide in the low salt buffer, 20 mM NaCl (Fig 2A lower panel). Overall, the shapes and breakpoints of these titrations were little affected by the change in salt concentrations, although the fluorescence increase was somewhat smaller than at high salt. These titrations suggest that the binding mode was similar at high and low ionic strengths, namely in the 65-70-base mode all across the titrations. The one significant change was in the shape of the curve with poly(dT) at close to saturation, possibly due to rearrangement along the ssDNA.

### Association kinetics with excess DNA over DCC-PfSSB

**Fig 3:**
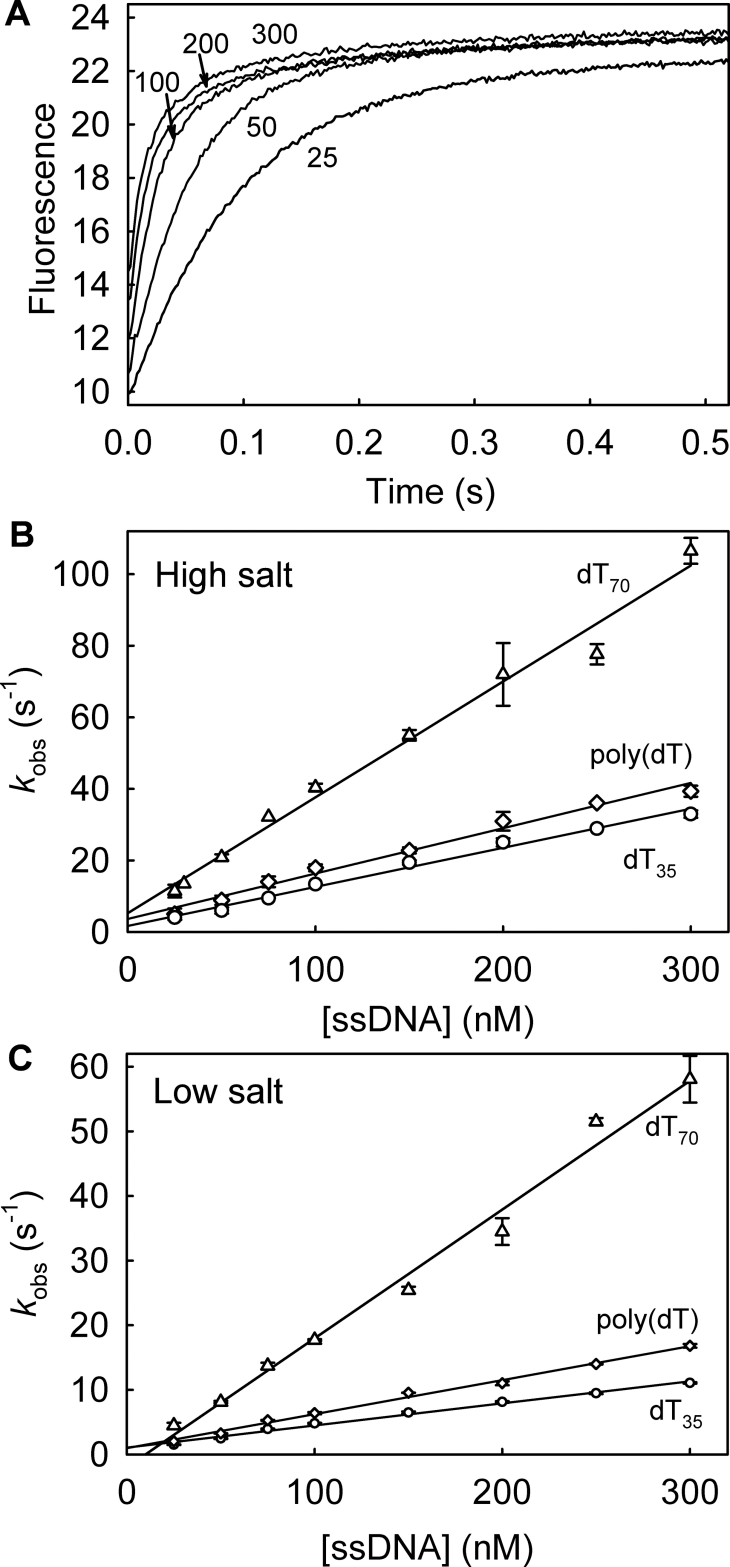
DCC-PfSSB association kinetics with excess ssDNA. (A) Fluorescence time courses of 5 nM DCC-PfSSB following mixing with dT_70_ in large excess and in high salt buffer. dT_70_ was at the nanomolar concentrations shown. (B) The data were fit to double exponentials: the fast rate constant increased linearly with concentration, giving an association rate constant of 324 ± 14 μM^-1^s^-1^ for dT_70_. Measurements with dT_35_ gave 127± 3 μM^-1^s^-1^ and 137 ± 6 μM^-1^s^-1^ for poly(dT). The intercept was too close to zero to give an accurate measure of the dissociation rate constant. (C) Association kinetics of dT_70_, dT_35_ and poly(dT) as in B but in low salt conditions. Time courses were fit to double exponentials. The association rate constants, determined from the linear fits of the fast phase, were 199 ± 9 μM^-1^s^-1^ for dT_70_, 34 ± 1 μM^-1^s^-1^ for dT_35_, and 53 ± 2 μM ^1^s^-1^ for poly(dT).

The fluorescence change on binding DCC-PfSSB to ssDNA was used to measure the association kinetics under pseudo-first order conditions. The fluorescence time courses were followed on a stopped-flow apparatus upon mixing DCC-PfSSB with a large excess of various lengths of ssDNA (Fig 3A). With dT_70_, the time courses were biphasic. The first, large phase (>80% of the total amplitude) had observed rate constants that linearly increased with concentration (Fig 3B). This gave a second-order association rate constant of 324 μM^-1^s^-1^. A small slow phase was also present with a rate constant (~2 s^-1^) that did not vary significantly with concentration and so may be a result of rearrangement of dT_70_, on PfSSB, for example.

The dT_35_ binding time courses (Panel A in S1 Fig) were well fitted by single exponentials indicating only a single process of binding being kinetically distinguishable. The observed rate constant increased linearly with concentration, giving the association rate constant of 109 **μ**M^-1^s^-1^ (Fig 3B). Similarly, the poly(dT) binding time courses (Panel B in S1 Fig) were well fitted by a single exponential, giving an association rate constant as 137 μM^-1^s^-1^. When the measurements were repeated with the three different DNA lengths under the low salt conditions (Fig 3C), the association rate constants were 2–4 fold lower than at high salt. However, all concentration dependences for the three lengths are linear, suggesting no change in binding rate constant in the concentration range tested.

### Association kinetics with excess DCC-PfSSB over DNA

**Fig 4:**
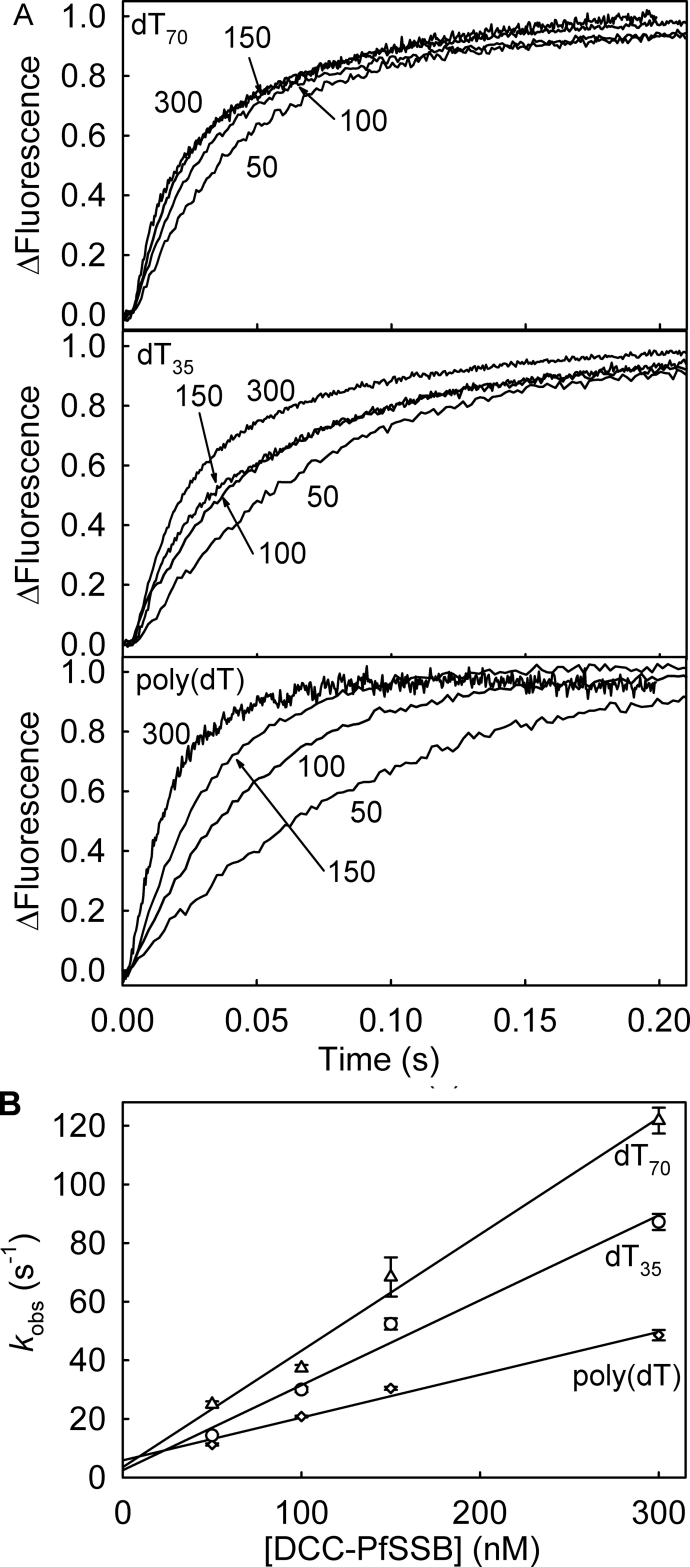
Association kinetics of ssDNA with excess DCC-PfSSB. The measurements were in high salt buffer with the ssDNA at 10% of the SSB concentration. (A) Example time courses for with DCC-PfSSB at the micromolar concentrations shown with dT_70_, dT_35_ and poly(dT) at 10-fold less concentration. To show on single panels traces are normalized to a total fluorescence change of 1. (B) Linear dependence of the observed rate constant of the fast phase on the concentration of the DCC-PfSSB, giving second order rate constants for dT_70_, 397 ± 31 μM^-1^s^-1^, poly(dT) 146 ± 13 μM^-1^s^-1^ and dT_35_ 290 ± 28 μM^-1^s^-1^.

The association kinetics of DCC-PfSSB and ssDNA were also measured with excess DCC-PfSSB over ssDNA (Fig 4). The time courses with dT_70_, dT_35_ and poly(dT) were biphasic. The large fast phase had rate constants that increased linearly with DCC-PfSSB concentration (Fig 4A and 4B) and with dT_70_ and poly(dT) gave second order rate constants similar to the association rate constants observed with excess ssDNA. A small slow phase did not change rate significantly with concentration with ~20% the amplitude of the fast phase.

The measurements with dT_35_ gave a second order rate constant of 264 μM^-1^s^-1^ (Fig 4A and 4B). Under these conditions, presumably only one dT_35_ binds to each PfSSB tetramer: this rate constant is ~2-fold faster than that obtained with excess dT_35_, where the transient represents binding of two molecules of dT_35_ to each tetramer.

Binding measurements with poly(dT) and excess protein showed time courses that were different from all other binding measurements. The traces were biphasic with a fast phase that increased linearly with concentration, but the slow phase was a decrease in fluorescence (Fig 4A), qualitatively similar to the time courses with DCC-EcSSB. The second phase had a rate constant of <2 s^-1^ at all concentrations and may be a slow rearrangement of PfSSB on poly(dT): this will be discussed later.

### DCC-PfSSB dissociation kinetics from ssDNA

**Fig 5:**
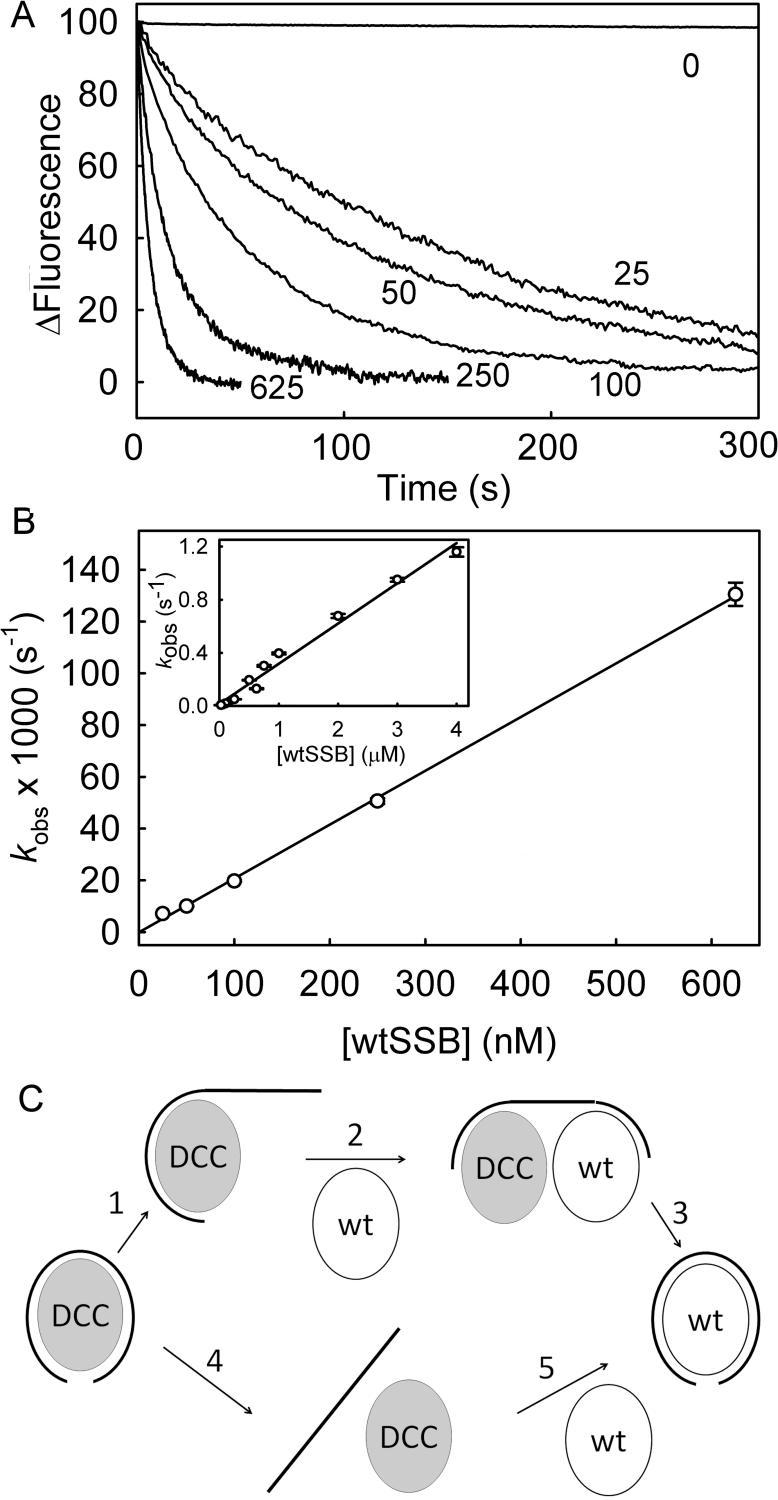
Dissociation kinetics for DCC-PfSSB.dT_70_. (A) The measurement were done in high salt buffer by premixing DCC-PfSSB, with a slight excess of dT_70_ to form the DCC-PfSSB·ssDNA complex before mixing with varying concentrations of wtSSB and following the fluorescence time course. The final concentrations in the reaction mix were 10 nM DCC-PfSSB, 12.5 nM dT_70_ and wtSSB at the nanomolar concentrations shown. (B) Dependence of dissociation kinetics on the wtSSB concentration. Time courses were fit to single exponentials and a linear fit of observed rate constants (*k*_obs_) gave the y intercept, -0.0001 ± 0.0009 s^-1^ and the gradient as 0.208 ± 0.003 μM^-1^s^-1^. The observed rate constants remain linearly dependent on [wtSSB] at higher concentrations (inset). (C) Model for mechanism of dissociation with either an associative pathway whereby wtSSB binds to the DNA before DCC-PfSSB dissociates (upper) or simple dissociation (lower). Steps are numbered so the forward and reverse rate constants for step *i* are *k*_+i_ and *k*_-I_, respectively.

DCC-PfSSB dissociation from ssDNA was measured by first forming the DCC-PfSSB·ssDNA complex before mixing it with large excess of the unlabeled wild-type PfSSB (wtSSB) to bind dissociated DNA. Wild type PfSSB has a higher affinity to ssDNA than DCC-PfSSB, as shown by the titration assays with equimolar labeled and unlabeled PfSSB (Fig 2B) So dissociated ssDNA binds almost quantitatively to the wild-type protein, following dissociation from its complex with DCC-PfSSB, observed as a drop in fluorescence (Fig 5).

If the DCC-PfSSB.ssDNA dissociation takes place as a single step and binding to the wild-type PfSSB trap is fast, the observed time courses would be a single exponential decrease that should not be affected by the concentration of the wtSSB. When the dissociation was measured with dT_70_, the fluorescence time courses became faster with increasing wild-type PfSSB concentration (Fig 5). These traces were fitted to a single exponential with a rate constant that was linearly dependent on the concentration of wtSSB (Fig 5A and 5B). The observed rate constant remained linearly dependent on wtSSB up to 4 μM (Fig 5B). This indicated that the dissociation is a complex process that may include interactions of the wtSSB with the DCC-PfSSB.dT_70_, as described for EcSSB [8]. The implications of this on determining a dissociation rate constant are described later.

The dissociation measurement was repeated in the low salt buffer, giving qualitatively similar result in that the response shows a linear increase in rate constant with wtSSB concentration (S3 Fig) The intercept () suggests the “true” dissociation rate constant is 0.014 s^-1^. This in turn gives, using the association rate constant of 199 μM^-1^s^-1^ obtained earlier a dissociation constant of 70 pM.

### AddAB helicase assay of linear dsDNA unwinding, using DCC-PfSSB

**Fig 6:**
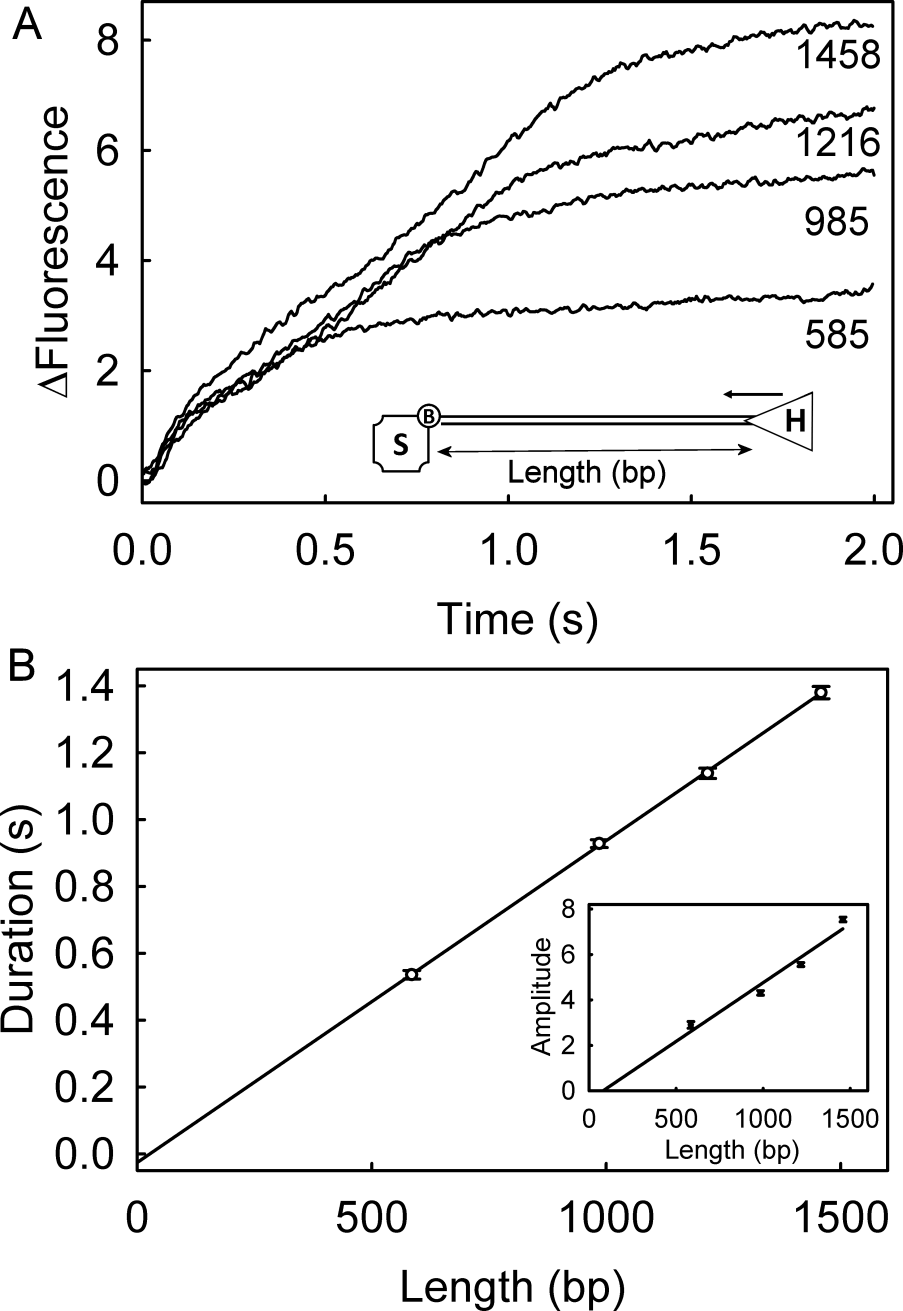
AddAB helicase assay with linear dsDNA using DCC-PfSSB as a biosensor. (A) Example fluorescence traces of AddAB different dsDNA lengths (shown in base pairs) using DCC-PfSSB as a reporter for production of ssDNA. The inset shows a cartoon of the complex before unwinding: S is streptavidin; B, biotin; H, helicase. (B) Linear fit of unwinding durations, plotted against the dsDNA lengths. The fit for the durations has gradient of 0.92 ± 0.02 s kb^-1^, which gives an unwinding rate of 1082 bp s^-1^. The inset shows the fluorescence changes plotted against dsDNA lengths.

DCC-PfSSB was tested in a helicase assay, namely AddAB-catalyzed unwinding of linear dsDNA, with a biotin at one end to allow that end to be blocked by streptavidin and leave only the distal end free for AddAB binding. The DNA was free of Chi sequences, which would otherwise cause a change in AddAB activity. AddAB was pre-incubated with the dsDNA to allow binding at the free blunt end, before rapidly mixing the complex with ATP in presence of DCC-PfSSB: the starting complex is shown diagrammatically in Fig 6A. The assay was done in the presence of a large excess of AddAB variant, AddA^*H*^B, that still binds to dsDNA ends/ssDNA, but has no helicase activity [22]. This acted as a trap, preventing active AddAB rebinding to dsDNA, once the reaction has started.

With all dsDNA lengths, a monotonic increase in fluorescence was observed, followed by relatively constant fluorescence (Fig 6). The duration of increasing fluorescence was dependent on the length of the dsDNA and represents AddAB unwinding dsDNA that leads to increasing amounts of ssDNA, to which the DCC-PfSSB binds. Once all the AddAB.dsDNA had unwound, the trap prevented further reaction. The unwinding duration increased linearly with dsDNA length (Fig 6B) and the unwinding rate was determined as 1082 bp s^-1^ from the reciprocal of the gradient of the fit. This is similar to results obtained previously under similar conditions [5].

## Discussion

### Choice of SSB-fluorophore adduct

In this study, single cysteines were positioned at various points on the SSB tetramer surface near but not within the DNA binding site. This ensured that one fluorophore could be placed on each subunit of the tetramer. Out of several positions tested, diethylaminocoumarin attached to C93, which is also the only native cysteine in wild-type PfSSB, produced the largest fluorescence change of our survey, while maintaining suitable properties to use this adduct as a probe for ssDNA. This particular adduct is termed DCC-PfSSB and was first used to characterize its interaction with ssDNA.

### ssDNA binding properties of DCC-PfSSB

By using the fluorescently labeled protein to provide a signal for binding, PfSSB shows considerable similarity to EcSSB in its kinetic mechanism for interaction with ssDNA, even though the crystal structure shows that DNA binds with opposite polarity [13]. Titrations (Fig 2) show that even at 125 pM DCC-PfSSB binding is stoichiometric, suggesting a dissociation constant of a few picomolar. This binding is too tight to quantify accurately by this equilibrium measurement, hence an alternate method was used to obtain the dissociation constant from the association and dissociation kinetics.

The binding is fast, possibly partially diffusion controlled. The observed rate of binding increases linearly with concentration (Figs 3 and 4), suggesting that a single step binding mechanism occurs. At all lengths of ssDNA tested, the association rate constant is somewhat slower at low salt conditions.

There is some variation in association rate constants depending on solution conditions and DNA length (Fig 3). The faster rate constant with dT_70_ relative to dT_35_ may directly relate to the length with these relatively unfolded oligonucleotides. However, poly(dT) is likely to be partially folded in solution and, given that concentration are shown in terms of 70-base lengths, the effective concentration available as target for SSB binding, is reduced. In all cases, either all or most of the fluorescence signal on binding is a single exponential. This suggests that, once part of the DNA has bound to the SSB in the second order process, the rest wraps rapidly in a first order process. This wrapping is not rate limiting so is not seen as a separate process in the binding signal.

### Dissociation of ssDNA

Unsurprisingly, dissociation is more complex than association (Fig 5), as was also seen with DCC-EcSSB [8]. Given the very tight binding with DCC-PfSSB, it was not possible to have conditions where ssDNA dissociates from its complex and remains dissociated. So to facilitate net dissociation from the complex, wild-type PfSSB at relatively high concentration was used. The observed rate constant for dissociation of DCC-PfSSb.dT_70_ was linearly dependent on the concentration of wtSSB (Fig 5). However, the time course of dissociation with high concentrations of the wild-type PfSSB could presumably be described in a similar way to DCC-EcSSB [8].

A mechanism for dissociation was considered with two distinct pathways (Fig 5C) in order to justify this observed linear dependence. In the associative pathway, as the ssDNA begins to unwrap from the DCC-*Pf*SSB.ssDNA complex (step 1), wtSSB can bind to the free end of DNA (step 2). This is followed by dissociation of the labeled SSB to give the final wtSSB.ssDNA complex (step 3). Assuming step 2 is slower than steps 1 and 3 and is reversible, the observed rate constant for this pathway is *k*_+2_[wtSSB]*k*_+3_(*k*_-2_+*k*_+3_). At very low concentrations of wtSSB, this pathway is less favoured and the simple dissociative pathway may occur, in which complete dissociation of DCC-*Pf*SSB.ssDNA (step 4) occurs before wtSSB binds to the ssDNA (step 5). Because the concentration of wtSSB is much greater than the labeled SSB, it may be assumed that step 4 is rate limiting and irreversible, so the observed rate constant is *k*_+4_. Overall, the observed rate constant for dissociation is, therefore, *k*_+4_ + *k*_+2_[wtSSB]*k*_+3_(*k*_-2_+*k*_+3_) and the y-intercept in Fig 5B gives the true dissociation rate constant, *k*_+4_. Because of the tight binding and large signal change with DCC-PfSSB, this measurement could be done at very low concentrations to give a more precise extrapolation to this intercept (Fig 5B inset). Even so, the intercept was too close to zero, so an approximate upper limit on the dissociation rate constant was obtained by considering the standard error on the intercept (-0.0001 ± 0.0009 s^-1^). From this, the 95% confidence limit gives the range for *k*_+4_ from -0.0017 to 0.0015, so the dissociation rate constant as likely to be <0.0015 s^-1^.

Based on this calculation and using the association rate constant, the equilibrium dissociation constant for dT_70_ and DCC-PfSSB is <4.6 pM. Labeling PfSSB reduces its affinity up to ~25-fold as shown by the titrations with a mixture of unlabeled and labeled PfSSB (Fig 2B), so the dissociation constant for unlabeled PfSSB is calculated as <200 fM.

The titrations support the binding capacity of each tetramer being 65–70 bases. This is shown by the poly(dT) titrations, where the fluorescence increases then plateaus at this ratio of poly(dT) bases to DCC-PfSSB (Fig 2A). When dT_35_ was titrated, two bound per tetramer, albeit with slightly different fluorescence enhancements (Fig 2A). This is not surprising, as the first dT_35_ can bind in the thermodynamically most favourable position. Given that the fluorophore does interfere with binding (25-fold lower affinity, described above), the first dT_35_ will preferentially bind away from the fluorophore. However, the second dT_35_ must fill the remainder of the binding groove. Overall, the three lengths of ssDNA give similar fluorescence enhancement, ~20-fold, on saturation.

### A large fluorescence enhancement

Having summarized basic binding of ssDNA, properties of DCC-PfSSB will be discussed that differ from DCC-EcSSB in terms of fluorescence and DNA binding. This includes evidence that 65-70-base binding mode is more favoured over 35-base binding with DCC-PfSSB, rather than DCC-EcSSB. In other words even at low salt conditions are possible where there is not a change in binding mode as described below.

DCC-PfSSB has a very high fluorescence enhancement on binding ssDNA (20-fold), ~3-fold greater change than DCC-EcSSB (6-7-fold). Diethylaminocoumarin fluorescence depends on its physical environment, in particular on the extent to which the diethylamino moiety is held coplanar to the rings [28–30]. Here, the change in environment is likely to be the DNA binding itself. The structure [13] is very similar to that of EcSSB, for which there is very little protein structural change, when DNA binds [14, 15]. The fluorescence quantum yields give an absolute measure of fluorescence. DCC-PfSSB in the absence of DNA has a quantum yield of 0.01, significantly lower than DCC-EcSSB (0.03) [8] or MDCC-PBP (also 0.03), which has the same fluorophore attached to the phosphate binding protein [25]. In all these cases, the quantum yield increases on ligand binding. The quantum yield of DCC-PfSSB is also lower than the diethylaminocoumarin in free solution as the IDCC adduct with a small thiol. Thus, it is likely that in uncomplexed DCC-PfSSB, the coumarin binds on the surface with the diethylamino group held out of the ring plane, so reducing the quantum yield relative to the free molecule. This allows for the very high signal change on DNA binding, when the coumarin diethylamino group and ring structure presumably become much more coplanar, as was observed in the crystal structure for the same fluorophore attached to a phosphate binding protein [28].

### Preferentially ssDNA fills all the binding groove

An important point with PfSSB is the extent to which its binding is mainly in the 65-base mode, so simplifying interaction with ssDNA under a range of conditions [11]. As described in the introduction, EcSSB has multiple binding modes, which result in different stoichiometries, depending on concentrations, ratio of ssDNA to SSB and salt concentration, in particular. In order to use this type of adduct as a biosensor, a single binding mode would greatly simplify experimental design and interpretation. A number of measurements with DCC-PfSSB gave evidence for this single binding mode predominating, even in low salt conditions, where EcSSB favors 35-base binding. This is in agreement with previous work on PfSSB [11].

Firstly, the binding stoichiometry of DCC-PfSSB to ssDNA is not greatly affected by the ionic strength of the buffer. This can be observed with all the three lengths of ssDNA tested (Fig 2), albeit the signal change is somewhat smaller in the low salt buffer. Especially, this is visible with both dT_70_ and dT_35_ titrations. With dT_70_ at both high and low salt, the titrations are monotonic, linear responses up to 1:1 ratio, not showing any curvature at low salt that DCC-EcSSB exhibited due to 35-base binding at low ratio. With dT_70_, the difference in binding of the first and the second DNA molecule is not much changed by the reduction of the ionic strength. In addition, the difference in fluorescent response between binding the first and the second dT_35_ is only small and both portions of the titration are linear, suggesting that both first and second DNA molecules bind tightly in both conditions. With DCC-EcSSB there were distinct phases for binding of the first and the second dT_35_ at both high and low salt, suggesting weak binding of the second particularly at low salt [4].

The effect of PfSSB:ssDNA ratio on binding mode was tested by measuring binding kinetics with high or low SSB. At low DCC-PfSSB and excess DNA, all binding time courses had a large rapid phase that got faster with concentration (Fig 3). A small slow phase had relatively constant kinetics, suggesting a first-order structural rearrangement. These features were basically similar to DCC-EcSSB [4]. In contrast as described in the introduction, association of DCC-EcSSB at high SSB in excess over ssDNA shows some distinct differences: there was a second, slow phase with a decrease in fluorescence in all cases [8]. Here with DCC-PfSSB, such a decrease was only observed with poly(dT) and it was lower in amplitude than with DCC-EcSSB. This suggests that the origin of this second phase may be interactions between SSB tetramers when bound together along the DNA.

Overall, our data suggest that PfSSB has a much greater preference for 65-70-base binding modes than does EcSSB. There is no direct evidence in our work why that is so, but as described above, PfSSB binds ssDNA very tightly (dissociation constant ~200 fM) in this mode, significantly tighter than EcSSB (2.6 nM by titration [4]). This may provide a simple thermodynamic basis for the preference: there would need a larger energetic change to move to 35-base binding. Kozlov *et al*. (2015) provided evidence that structural, sequence and size difference between EcSSB and PfSSB cooperativity domains could be the reason for different DNA binding modes between these species [12]. AS described in the Introduction, the disordered C-terminus is likely to play an essential role in cooperative binding, which is mainly a factor in 35-base binding. Because this binding mode is greatly reduced with Pf-SSB, then cooperative binding is less of a factor. In itself, cooperative binding is not deleterious to use of DCC-PfSSB as mainly the rate of fluorescence change is limited by the rate of ssDNA formation. It is only when the binding mode changes during an assay or series of assays that this may become a factor in interpreting the fluorescence signal. However, we have shown here that this is unlikely with DCC-PfSSB.

### Advantages of using *Plasmodium* SSB in a biosensor

DCC-PfSSB has provided a route to get information about DNA binding properties of PfSSB. However, a major impetus to characterizing this adduct was to investigate its potential as a biosensor to follow ssDNA formation in real time, especially by helicases. DCC-PfSSB has three main potential advantages over EcSSB for this use. At ~20-fold, its signal change on fully binding ssDNA is ~3-fold greater than with the *E*. *coli* protein (Fig 1). Secondly, the fluorescence response is linear over a wide range of conditions, low and high salt and at very low concentrations (Fig 2). In particular, assays of ssDNA formation are likely to use conditions equivalent to the early part of these titrations, in which DCC-PfSSB is in significant excess over ssDNA being formed. The third advantage is that the binding occurs in a single mode of a wider range of conditions, making the interpretation of the fluorescence data much simpler. As an example, a DNA unwinding assay was performed under conditions that previously indicated a change in binding mode of DCC-EcSSB as the reaction progressed [5]. With DCC-EcSSB, this change resulted in a decrease in fluorescence at longer time: with DCC-PfSSB, there was no such reduction in fluorescence (Fig 6).

In summary, the diethylaminocoumarin labeling of *Plasmodium* SSB provides an improved and sensitive tool to measure ssDNA, taking advantage of the simple binding under a range of conditions. The background fluorescence is low and the signal change is large with a linear response as DNA becomes saturated, so that assays can be performed at low concentrations.

## Supporting information

S1 Fig Fluorescence time courses for DCC-PfSSB binding to excess ssDNA. Representative set of time courses for (A) dT35 or (B) polydT in high salt conditions. (C) dT_70_, (D) dT_35_ or (E) polydT in low salt conditions The experiments were done as in Fig 3 and the concentrations of DNA are shown in nanomolar.
(PDF)

S2 Fig Fluorescence time courses for ssDNA binding to excess DCC-PfSSB binding. Traces were obtained as in Fig 4. The data show long times for (A) dT_70_, (B) dT_35_ and (C) polydT in low salt conditions.
(PDF)

S3 Fig DCC-PfSSB dissociation kinetics from dT70 in low salt conditions. DCC-PfSSB was premixed with a slight excess of dT_70_ to form the DCC-PfSSB·ssDNA complex before mixing with varying concentrations of wtSSB and following the fluorescence time course. Experimental conditions, except for salt concentration, were as in Fig 5. (A) Individual traces of at various wtSSB concentrations, shown in micromolar. (B) These traces were fitted to single exponentials to determine an observed rate constant (*k*_obs_) at each wtSSB concentration. These were plotted against [wtSSB]. The linear fit had a gradient of 0.107 ± 0.008 μM^-1^s^-1^ and intercept of 0.014 ± 0.004 s^-1^.
(PDF)

S1 Table List of primers used for preparing mutant DNA. These were used to change the sequence from the original synthesized gene for (G103C)PfSSB.
(PDF)
