# Supplementary material for: Fluorescent single-stranded DNA-binding protein from Plasmodium falciparum as a biosensor for single-stranded DNA
Source: PLoS One. 2018 Feb 21;13(2):e0193272. doi: 10.1371/journal.pone.0193272 (PMC5821389; doi:10.1371/journal.pone.0193272)

S1 Fig. Fluorescence time courses for DCC-PfSSB binding to excess ssDNA. Representative set of time courses for (A) dT_{35} or (B) polydT in high salt conditions. (C) dT_{70}, (D) dT_{35} or (E) polydT in low salt conditions The experiments were done as in Fig 3 and the concentrations of DNA are shown in nanomolar.

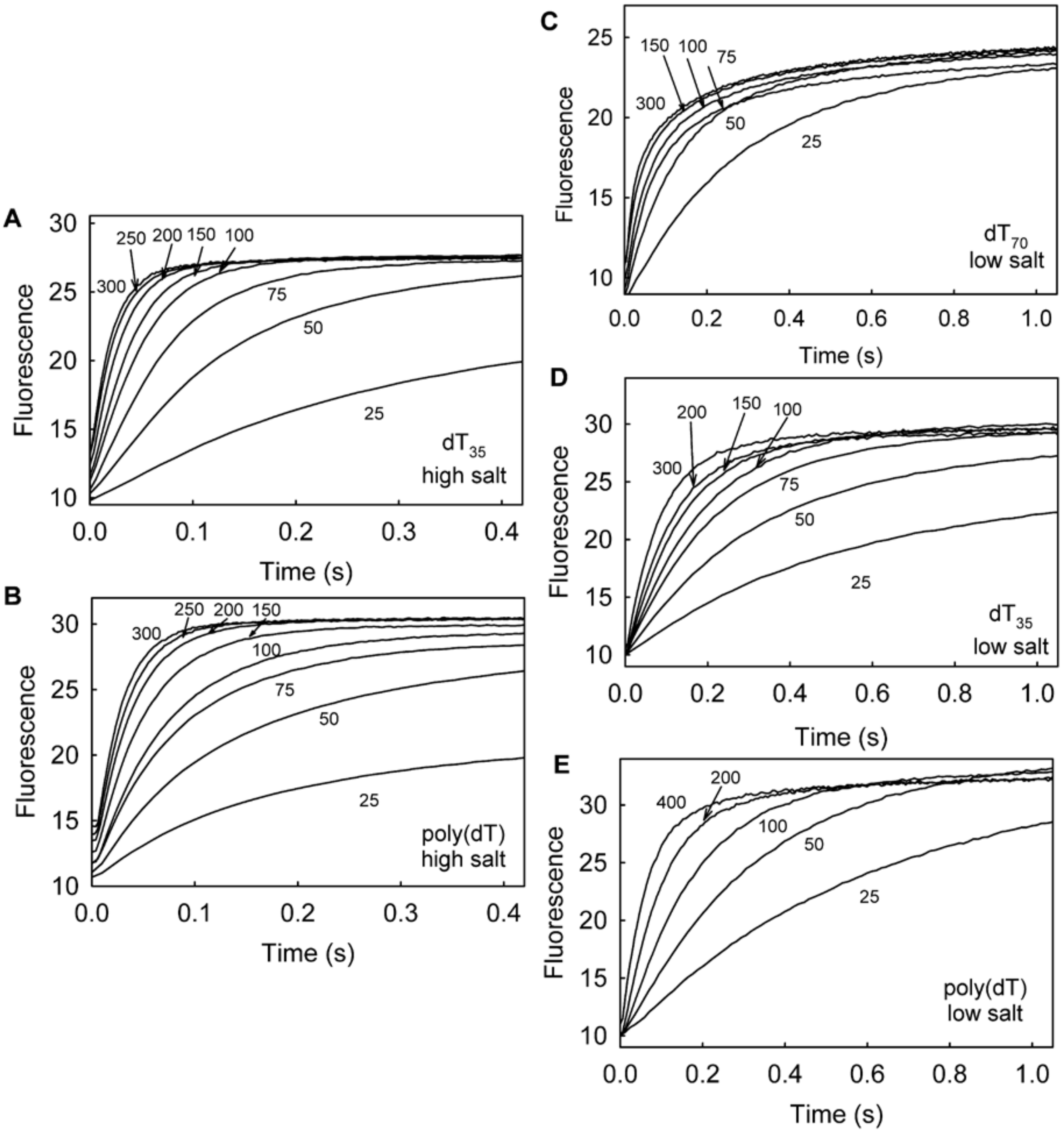

Supplement: S1 Fig — Representative set of time courses for (A) dT35 or (B) polydT in high salt conditions. (C) dT70, (D) dT35 or (E) polydT in low salt conditions The experiments were done as in Fig 3 and the concentrations of DNA are shown in nanomolar. (PDF) [file pone.0193272.s001.pdf]
