# Supplementary material for: Fluorescent single-stranded DNA-binding protein from Plasmodium falciparum as a biosensor for single-stranded DNA
Source: PLoS One. 2018 Feb 21;13(2):e0193272. doi: 10.1371/journal.pone.0193272 (PMC5821389; doi:10.1371/journal.pone.0193272)

S2 Fig. **Fluorescence time courses for ssDNA binding to excess DCC-PfSSB binding.** Traces were obtained as in Fig. 4. The data show long times for (A) dT_{70}, (B) dT_{35} and (C) polydT in low salt conditions.

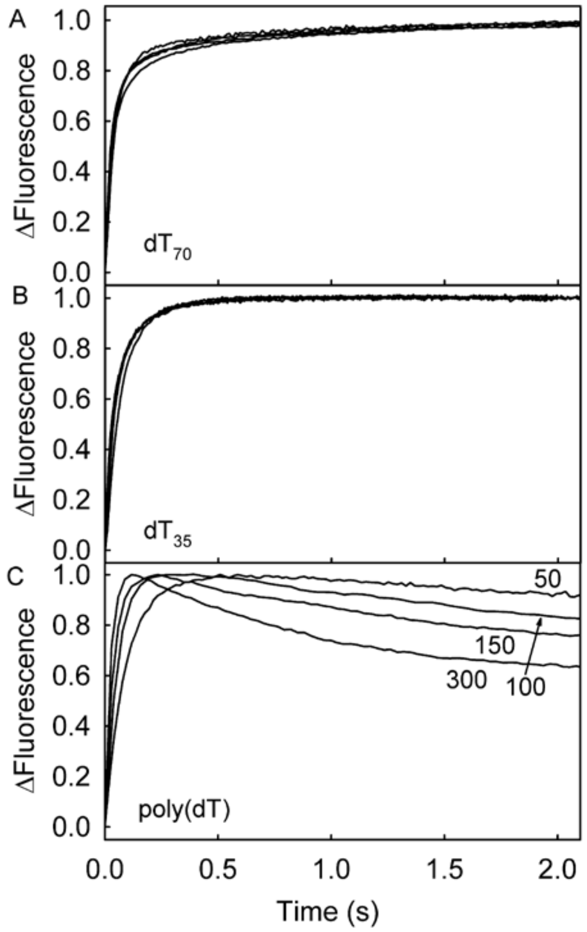

Supplement: S2 Fig — Traces were obtained as in Fig 4. The data show long times for (A) dT70, (B) dT35 and (C) polydT in low salt conditions. (PDF) [file pone.0193272.s002.pdf]
