# Supplementary material for: Fluorescent single-stranded DNA-binding protein from Plasmodium falciparum as a biosensor for single-stranded DNA
Source: PLoS One. 2018 Feb 21;13(2):e0193272. doi: 10.1371/journal.pone.0193272 (PMC5821389; doi:10.1371/journal.pone.0193272)

S3 Fig. **DCC-PfSSB dissociation kinetics from dT_{70} in low salt conditions.** DCC-PfSSB was premixed with a slight excess of dT_{70} to form the *DCC*-PfSSB·ssDNA complex before mixing with varying concentrations of wtSSB and following the fluorescence time course. Experimental conditions, except for salt concentration, were as in Fig 5. (A) Individual traces of at various wtSSB concentrations, shown in micromolar. (B) These traces were fitted to single exponentials to determine an observed rate constant (k_{obs}) at each wtSSB concentration. These were plotted against [wtSSB]. The linear fit had a gradient of 0.107 ± 0.008 $\mu M^{-1} s^{-1}$ and intercept of 0.014 ± 0.004 s^{-1}

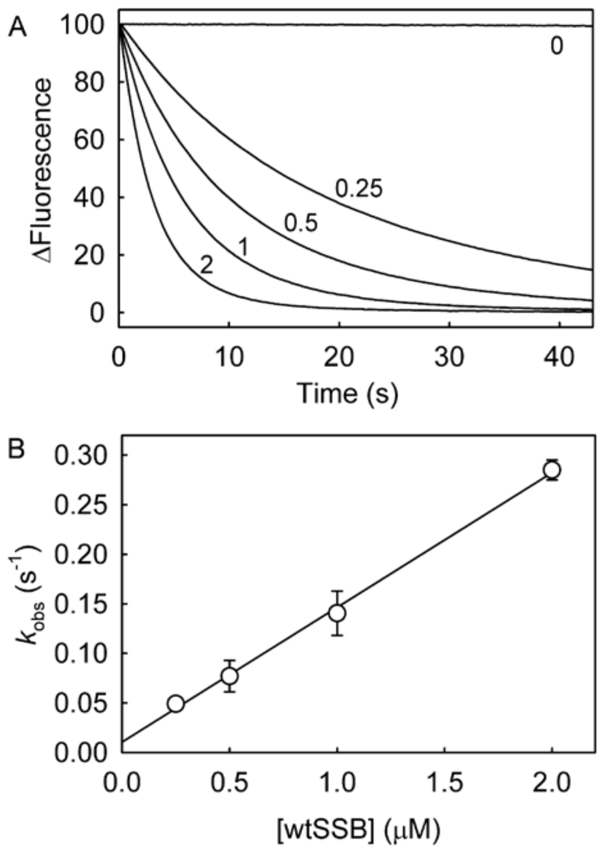

Supplement: S3 Fig — DCC-PfSSB was premixed with a slight excess of dT70 to form the DCC-PfSSB·ssDNA complex before mixing with varying concentrations of wtSSB and following the fluorescence time course. Experimental conditions, except for salt concentration, were as in Fig 5. (A) Individual traces of at various wtSSB concentrations, shown in micromolar. (B) These traces were fitted to single exponentials to determine an observed rate constant (kobs) at each wtSSB concentration. These were plotted against [wtSSB]. The linear fit had a gradient of 0.107 ± 0.008 μM-1s-1 and intercept of 0.014 ± 0.004 s-1. (PDF) [file pone.0193272.s003.pdf]
