# Supplementary material for: Fluorescent single-stranded DNA-binding protein from Plasmodium falciparum as a biosensor for single-stranded DNA
Source: PLoS One. 2018 Feb 21;13(2):e0193272. doi: 10.1371/journal.pone.0193272 (PMC5821389; doi:10.1371/journal.pone.0193272)
Supplement: S1 Table — These were used to change the sequence from the original synthesized gene for (G103C)PfSSB. (PDF) [file pone.0193272.s004.pdf]

S1 Table. List of primers used for preparing mutant DNA. These were used to change the sequence from the original synthesized gene for (G103C)PfSSB.

Mutation		Sequence
C103G	Forward	CCTGAACGGCGGTGACAAAGTTGCGACCTTTAGCC
	Reverse	GCAACTTTGTCACCGCCGTTCAGGATTTTAATGTCCG
C93A	Forward	GTCGCGTGGGCGCTGAACCGGACATTAAAATCCTG
	Reverse	CTCCGGTTCAGCGCCCACGCGACCGATCAGC
G102C	Forward	AATCCTGAACTGCGGTGACAAAGTTGCGACCTTTAGC
	Reverse	CTTTGTCACCGCAGTTCAGGATTTTAATGTCCGG
W166C	Forward	GCATACCCGCAAATGCCACACGAACGACATGAATTCAC
	Reverse	GCAAGCACACCGTAAACGCCCATACGTCCCTTGGAAG
Y156R	Forward	GGCCGTCGCGTCAGAGTGCAAGGTTCCCTGCATACCCGC
	Reverse	GGGAACCTTGCACTCTGACGCGACGGCCTTTACGCAG
